# Integrative genomic and functional profiling of the pancreatic cancer genome

**DOI:** 10.1186/1471-2164-14-624

**Published:** 2013-09-16

**Authors:** A Hunter Shain, Keyan Salari, Craig P Giacomini, Jonathan R Pollack

**Affiliations:** 1 Departments of Pathology, Stanford University School of Medicine, 269 Campus Drive, CCSR-3245A, Stanford, CA 94305-5176, USA; 2 Department of Genetics, Stanford University School of Medicine, 269 Campus Drive, CCSR-3245A, Stanford, CA 94305-5176, USA

**Keywords:** Pancreatic cancer, Functional genomics, RNAi screen, shRNA screen, NUP153, KLF5

## Abstract

**Background:**

Pancreatic cancer is a deadly disease with a five-year survival of less than 5%. A better understanding of the underlying biology may suggest novel therapeutic targets. Recent surveys of the pancreatic cancer genome have uncovered numerous new alterations; yet systematic functional characterization of candidate cancer genes has lagged behind. To address this challenge, here we have devised a highly-parallel RNA interference-based functional screen to evaluate many genomically-nominated candidate pancreatic cancer genes simultaneously.

**Results:**

For 185 candidate pancreatic cancer genes, selected from recurrently altered genomic loci, we performed a pooled shRNA library screen of cell growth/viability across 10 different cell lines. Knockdown-associated effects on cell growth were assessed by enrichment or depletion of shRNA hairpins, by hybridization to barcode microarrays. A novel analytical approach (COrrelated Phenotypes for On-Target Effects; COPOTE) was used to discern probable on-target knockdown, based on identifying different shRNAs targeting the same gene and displaying concordant phenotypes across cell lines. Knockdown data were integrated with genomic architecture and gene-expression profiles, and selected findings validated using individual shRNAs and/or independent siRNAs. The pooled shRNA library design delivered reproducible data. In all, COPOTE analysis identified 52 probable on-target gene-knockdowns. Knockdown of known oncogenes (KRAS, MYC, SMURF1 and CCNE1) and a tumor suppressor (CDKN2A) showed the expected contrasting effects on cell growth. In addition, the screen corroborated purported roles of PLEKHG2 and MED29 as 19q13 amplicon drivers. Most notably, the analysis also revealed novel possible oncogenic functions of nucleoporin NUP153 (ostensibly by modulating TGFβ signaling) and Kruppel-like transcription factor KLF5 in pancreatic cancer.

**Conclusions:**

By integrating physical and functional genomic data, we were able to simultaneously evaluate many candidate pancreatic cancer genes. Our findings uncover new facets of pancreatic cancer biology, with possible therapeutic implications. More broadly, our study provides a general strategy for the efficient characterization of candidate genes emerging from cancer genome studies.

## Background

Pancreatic ductal adenocarcinoma (hereafter, pancreatic cancer) is the fourth leading cause of cancer death in the United States [1,2]. The five-year survival rate is a dismal 5%, as effective treatment regimens are limited [3]. A better understanding of the underlying disease biology is needed to develop new and successful treatment strategies to manage this deadly disease.

Several key molecular genetic alterations in pancreatic cancer have been identified [4,5]. Activating mutations of *KRAS* occur in 95% of cases. The *CDKN2A* locus, encoding p16INK4A and p14ARF, which respectively intersect the Rb and p53 pathways, is homozygously deleted in 80% of tumors. *TP53* is itself inactivated, usually through point mutation, in 55% of cases. *SMAD4*, a central mediator of TGFβ signaling, is deleted in approximately 50% of cases. Furthermore, *TGFBR2*, its upstream receptor, is deleted in 20% of tumors, underscoring a central importance of this signaling pathway in pancreatic cancer. *MYC* is amplified in approximately 30% of cases. Recently, deletions and mutations in five different subunits of the SWI/SNF chromatin remodeling complex have been found to occur in about a third of cases [6]. However, despite what is already known, recent surveys of the pancreatic cancer genome have identified scores of additional candidate cancer genes that merit further investigation [7,8].

With the advent of DNA microarrays and “next-generation” DNA sequencing, the field of genomics has transformed our ability to study diseases like cancer on an “omic” scale. Over the past decade, these technologies have spurred structural studies producing a compendium of cancer alterations, including DNA mutations, deletions, amplifications, and rearrangements. Yet, because of the sheer volume of data, such studies have far outpaced our ability to functionally evaluate candidate cancer genes [9].

The development of RNA interference (RNAi) techniques has accelerated our capacity to study knockdown phenotypes and infer the function and mechanism of disease genes [10]. While traditionally used to characterize single genes at a time, several groups have adapted the technology to use small interfering RNA (siRNA) or short hairpin RNA (shRNA) libraries for high-throughput screens [11], including in pancreatic cancer [12-17]. These large-scale, highly parallel efforts provide the potential to functionally annotate genes on an “omic” scale.

Here, we describe a high-throughput functional interrogation of the pancreatic cancer genome using an shRNA-based screen. We simultaneously evaluate 185 candidate pancreatic cancer genes, nominated from genomic profiles, across 10 genetically diverse cell lines. After integrating the functional and genomic data, we further characterize nine top candidates, both uncovering new pancreatic cancer biology and validating an integrative approach for the functional annotation of cancer genomes.

## Methods

### Cell lines

Cancer cell lines were obtained directly from the American Type Culture Collection, and grown in RPMI-1640 high-glucose media (Invitrogen) supplemented with 10% fetal bovine serum (Hyclone). HPDE cells [18] were obtained from Dr. Ming Tsao (University of Toronto), and grown in keratinocyte serum-free media (supplemented as directed with EGF and bovine pituitary extract; Invitrogen).

### Pooled shRNA lentiviral library screen

**Figure 1:**
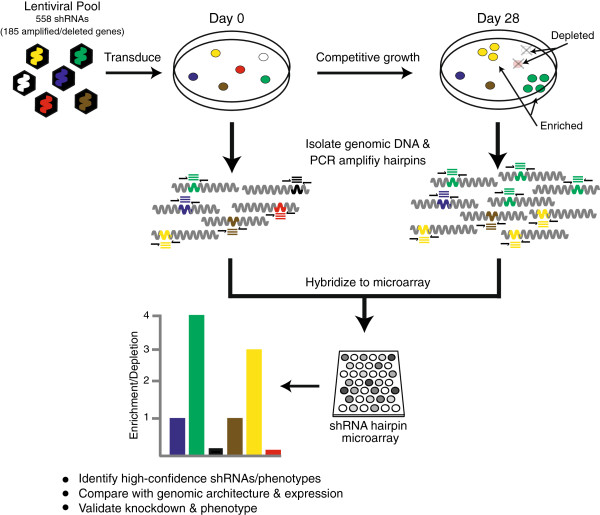
**Targeted shRNA screen.** Schematic depiction of the shRNA screen, analysis, and validation. See main text for detailed description.

The shRNA screen, schematically depicted in Figure 1 (with summary information in Table 1), was adapted from published protocols [19,20]. Potential advantages of a pooled (compared to well-based) screen include economies of scale and discernment of subtle fitness effects by competitive growth over many days. The 185 targeted genes were selected based on the identification of recurrent structural abnormalities (focal DNA amplifications, deletions, and/or mutations) in pancreatic cancer genomes [6,7]. These abnormalities are listed for each gene in Additional file 1. GIPZ lentiviral shRNAmir constructs targeting these genes (average 3, range 1–7 shRNA/gene) were obtained from Open Biosystems/Thermo Scientific; catalog numbers are listed in Additional file 2. The 558 pGIPZ shRNAmir plasmid DNAs were combined at equimolar concentration into a single pool. The shRNA DNA pool was then used to transfect 293T cells, together with a trans lentiviral packaging mix (Open Biosystems). Pooled shRNA lentiviral supernatant was collected 48 hrs later, and frozen in aliquots to improve screen reproducibility.

**Table 1:** Cell lines included in shRNA screen

**Cell line**	**Description**	**Number of replicate infections**	**Multiplicity of infection**	**shRNA fold-representation**
Aspc1	Pancreatic cancer	3	0.3	1,000
BXPC3	Pancreatic cancer	3	0.3	1,500
Capan1	Pancreatic cancer	7	0.3	400
HPAC	Pancreatic cancer	3	0.3	1,500
Panc1	Pancreatic cancer	6	0.3	150
PL5	Pancreatic cancer	3	0.3	2,000
SU86.86	Pancreatic cancer	3	0.3	1,000
HPDE	Immortalized, non-tumorigenic pancreatic ductal epithelial cells	3	0.3	1,000
MDA157	Breast cancer	3	0.3	600
SKBR3	Breast cancer	3	0.3	1,000

The lentiviral library was then used to infect target cell lines at low multiplicity of infection (average 0.3 integrants/cell; determined by flow cytometry of GFP expression from the GIPZ vector), so that most cells contained a single shRNA knocking down the expression of a single gene. Additionally, enough cells were infected to provide an average representation of approximately 1,000 lentiviral integrations (range 150–2000) for each of the 558 shRNAs in the library, mitigating potential artifacts from specific integration sites or from multiple integrations [20,21]. To infect target cell lines, lentivirus was diluted in serum/antibiotic-free media containing 10 μg/ml polybrene (determined to optimally enhance infection with minimal cell toxicity). Cells were spun at 30°C for 1 hr at 2,400 rpm, allowed to recover for 4 hrs (37°C, 5% CO_2_), spun again for an additional 1 hr, and then the media replaced with complete RPMI-1640 growth media. All target cell line infections were carried out minimally in triplicate (range 3–7 replicates).

Two days post infection, a fraction of the infected cells was harvested for an initial time point (T = 0), and puromycin selection (using cell line-specific levels previously determined by killing curves) was then initiated for the remaining cells. Cells were cultured for an additional 4 weeks in the presence of selective media. Care was taken not to allow the cells to become too confluent or to split too thinly. Cells were periodically harvested, including the last time point (T = 4 weeks), which was the standard comparison point for the screen data presented.

Genomic DNA was isolated from harvested cells, sheared ten times through a 25 gauge needle, and subsequently used as template for PCR amplification of library shRNA hairpins. Sufficient genomic DNA template was included in the PCR reaction to ensure an approximate 1,000-fold average representation of each library shRNA hairpin (assuming 5 pg of DNA/cell), thereby maintaining the initial ~1,000-fold representation of integrations. PCR primers common to the shRNAmir backbone vector and bounding the half hairpin target sequence were as follows: Forward 5′-TAGTGAAGCCACAGATGTA-3′; Reverse 5′-ATGTATCAAAGAGATAGCAAGGTATTCAG-3′.

To deconvolute shRNA representation in the T = 4 weeks *vs*. T = 0 cell pools, gel-purified PCR products were differentially labeled and co-hybridized (using manufacturer protocols) to a custom Agilent microarray designed (using Agilent eArray software) to include probes complementary to shRNA hairpin sequences. Microarrays were imaged and fluorescence intensities extracted. For each microarray, log_10_ background-subtracted fluorescence ratios were globally normalized (which assumes overall equal library shRNA depletion and enrichment). Unless otherwise specified, screen data reported represent the average ratios from replicate cell line infections.

### Screen data analysis and validation

High-confidence on-target shRNAs/phenotypes were identified using a new analytical approach (COrrelated Phenotypes for On-Target Effects; COPOTE), based on finding two or more different shRNAs targeting the same gene and displaying concordant enrichment/depletion profiles across the cell lines (Pearson correlation). Custom Perl scripts were used to calculate the Pearson correlation coefficient between shRNAs targeting the same genes, and also to permute cell line identities to generate the randomized null distribution of correlations. To define meaningful correlations, we determined False Discovery Rates (FDRs) as the ratio of false positives (from the null distribution) to observed positives at or above any given correlation cutoff.

For validation, Q-RT-PCR was performed using Assay-on-Demand TaqMan probes and reagents (Applied Biosystems). Catalog numbers for probes include: NUP153 (Hs01018919_m1) and KLF5 (Hs00156145_m1). Western blots were done on whole cell lysates, using the following primary antibodies: Myc (Santa Cruz sc-40), pan-Ras (Millipore 05–516), NUP153 (Acris BM5527), GAPDH (Santa Cruz sc-25778), SMURF1 (Santa Cruz H-60). Densitometry calculations were carried out using publicly available ImageJ software. For siRNA knockdown, ON-TARGETplus SMARTpool siRNAs were obtained from Thermo Scientific: KRAS (L-005069-00-0005), SMURF1 (L-007191-00-0005), NUP153 (L-005283-00-0005), KLF5 (L-013571-00-0005), non-targeting control (D-001810-10-20). Cell growth/viability assays were done using a modified WST-1 protocol as previously described [6]. In each assay, a full time course was performed, though usually only day 5 is shown for brevity.

### Array-based comparative genomic hybridization (aCGH) data

Findings from the screen were interpreted in the context of previously published aCGH data [6] (GSE26089). Briefly, that data set comprises Agilent 244 K CGH array profiles from 70 pancreatic cancers (48 primary tumor xenografts and 22 cancer cell lines). Tumor/normal fluorescence ratios were normalized and mapped onto the genome (build 18) using Agilent software.

## Results and discussion

### Targeted shRNA screen

In pancreatic (as with other) cancer genomes, loci that are recurrently amplified or deleted are likely enriched for known or novel cancer genes [22]. Typically, each such locus is studied individually to discover the driver gene(s). In an effort to accelerate this process, we developed a pooled shRNA screening strategy to simultaneously evaluate 185 candidate pancreatic cancer genes that together represent 104 different loci of recurrent DNA amplification or deletion; thus the genes selected included both candidate oncogenes and tumor suppressors (see Additional file 1). Most of the 185 candidates were focally amplified or deleted in a subset of cancers, and some were also reported to carry mutations (Additional file 1) [6,7]. A few known cancer genes (e.g. *KRAS*, *MYC*, and *CDKN2A*) were also included in the screen as positive controls.

The pooled shRNA screen was carried out as a competitive growth/viability assay. The general workflow, adapted from Schlabach *et al.*[19], is depicted in Figure 1 and described in more detail in Methods. In brief, pancreatic cancer cell lines were infected with a pooled shRNA lentiviral library comprising 558 shRNAs targeting the 185 genes (on average 3 hairpins/gene) (Additional file 1). Infections were done at low multiplicity of infection, ensuring that most cells harbored a single shRNA knocking down the expression of a single gene. The pooled, infected cells were then cultured for four weeks, after which depleted or enriched shRNAs (i.e., those targeting genes conferring positive or negative growth advantage, respectively) were identified by PCR amplification of shRNA hairpins and comparative hybridization (T = 4 weeks *vs*. T = 0) to a custom hairpin microarray.

The shRNA library screen was carried out on ten different cell lines (Table 1). These included seven genetically-diverse pancreatic cancer cell lines that together harbor the vast majority of copy number alterations from which the 185 genes were selected. We also included a single immortalized, non-tumorigenic human pancreatic ductal epithelial (HPDE) cell line [18] to facilitate discovery of tumor suppressor genes (as enriched shRNAs targeting growth suppressive genes). Lastly, we also screened two breast cancer cell lines to help distinguish genome-specific from generic essential genes (i.e., an “out-group”). The raw and cell line-averaged screening data are available in Additional files 2 and 3.

### Screen analysis and validation

**Figure 2:**
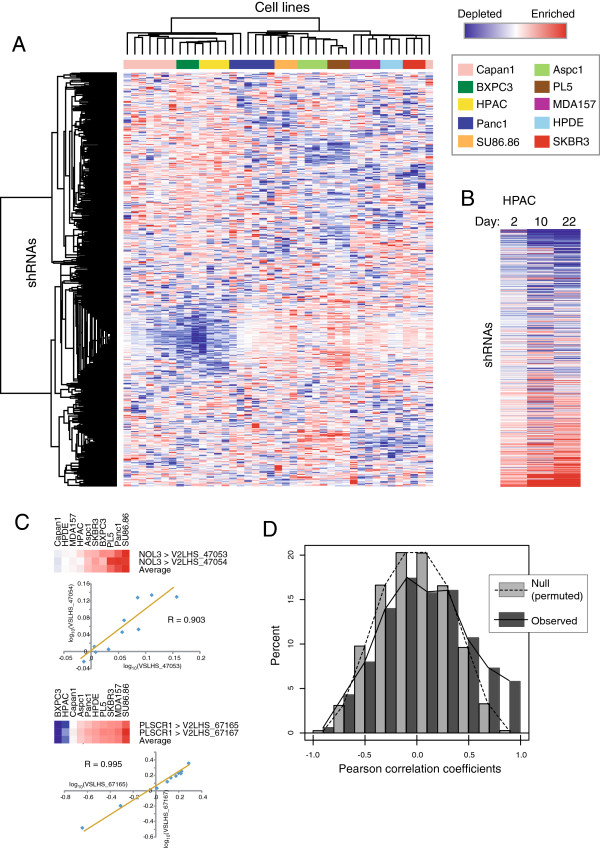
**Performance of shRNA screen. (A)** Heatmap representation of unsupervised hierarchical cluster analysis of screen data. Columns represent screened cell lines (key provided), and rows represent the 558 different shRNAs (here, mean-centered). The color scale indicates fold-depletion (blue) or fold-enrichment (red) of shRNAs in T = 4 week *vs*. T = 0 comparisons. Note that replicate screens for each cell line tend to cluster together, which demonstrates reproducibility across all steps of the screen (infection, cell line passaging, PCR amplification, and microarray hybridization). The basis for the single outlier (Capan1, *far right*) is unclear. **(B)** Time course analysis of shRNA depletion/enrichment, shown for a single cell line (HPAC). Note that across the time series, library shRNAs show expected increased depletion (blue, top of heat map) or enrichment (red; bottom of heat map), consistent with competitive growth selection. **(C)** Depletion/enrichment status of selected genes is shown. Knockdown of the same gene by different shRNAs often produced concordant phenotypes; e.g., Pearson correlation (R) = 0.903 for two hairpins targeting *NOL3*, and R = 0.995 for two hairpins targeting *PLSCR1*. **(D)** Frequency plot of observed Pearson correlations (R values; correlating depletion/enrichment vectors across the cell line panel) between hairpin pairs targeting the same gene (dark gray bars) compared to a null distribution of Pearson correlations generated from randomly permuted data (light gray bars). Note, the rightward shift of observed Pearson correlations, above that expected by chance, reflects enrichment of on-target shRNAs/phenotypes.

The pooled shRNA library screen yielded high quality and reproducible data, as supported by multiple lines of evidence. First, replicate screens of each cell line showed good correlation; the average pair-wise Pearson correlation coefficients (R-values) for cell line replicates ranged from 0.3-0.9 (mean 0.5). The two cell lines (Capan1 and Panc1) with the lowest R-values were those with the least shRNA library representation; however, this was mitigated by more screen replicates (Table 1). Notably, in an unsupervised analysis of the shRNA depletion/enrichment data, cell line replicates most often clustered together (Figure 2A). Second, time course samplings, done for a subset of the cell lines, demonstrated consistently increasing shRNA depletion (or enrichment) over time (Figure 2B). Third, we noted that distinct shRNAs targeting the same gene often exhibited correlated growth phenotypes (depletion/enrichment profiles) across the panel of cell line screened (Figure 2C). We capitalized on this last observation to define “high-confidence” on-target knockdown phenotypes, an approach we termed COPOTE (COrrelated Phenotypes for On-Target Effects).

When analyzing hundreds of shRNAs, it is possible that two different shRNAs targeting the same gene have a similar growth phenotype (across the panel of cell lines) just by random chance. Therefore, to correct for multiple hypothesis testing, we compared the distribution of observed Pearson correlations to that from randomly permuted data (Figure 2D). Notably, we observed a significant rightward shift in the observed distribution of correlations, indicating an enrichment of shRNAs targeting the same gene (and showing similar phenotype) above that expected by chance; false discovery rates (FDR; q values) are reported in Additional file 4. Of the 185 genes included in the screen (and of the 157 genes (85%) represented by two or more shRNAs and therefore evaluable), we identified 52 genes with “high confidence” knockdown phenotypes, defined by having at least two different shRNAs exhibited significantly correlated depletion/enrichment profiles (R > 0.7; q < 0.135) across the ten cell lines.

We note that other approaches have been used to leverage information from multiple shRNAs targeting the same gene, including redundant siRNA activity (RSA) analysis [23], RNAi gene enrichment ranking (RIGER) [24], and the Gene Activity Ranking Profile (GARP) score [17]. All of these methods consider either the top-most depleted shRNA(s) or all shRNAs for a given gene. Our approach is fundamentally different in that we consider all shRNA pairs, therefore focusing primarily on the evidence for correlated (implying on-target) phenotype rather than on the strength of shRNA depletion.

Focusing primarily on the 52 high-confidence genes, we next validated knockdown for a subset of biologically-interesting genes. We included some known pancreatic cancer genes, but mainly focused on novel genes with biological functions plausibly related to pancreatic cancer, and also demonstrating logical connections to the underlying genomic data, e.g. shRNA depletion for amplified candidate oncogenes. Validation was done by transducing individual (rather than the pool of) shRNAs, or by transfecting independent siRNAs (which we found to be more time and cost effective), and then verifying target knockdown by RT-PCR and/or Western blot (summarized in Table 2). For known pancreatic cancer oncogenes and tumor suppressor genes, finding the expected growth phenotypes (as described below) provided additional screen validation.

**Table 2:** Summary of validation for target knockdown and growth phenotype

**Gene**	**shRNA knockdown**	**shRNA knockdown**	**shRNA knockdown**	**siRNA knockdown**	**siRNA knockdown**	**siRNA knockdown**
	**validated by RT-PCR**	**validated by**	**validated by cancer**	**validated by**	**validated by**	**growth phenotype**
		**western blot**	**genome anatomy**	**RT-PCR**	**western blot**	**validated**
			**Project**[25]			
*KRAS*		X [26]		X	X	X
*SMURF1*		X			X	X
*CDKN2A*			X			
*CCNE1*			X			
*NUP153*	X				X	X
*KLF5*		X		X	X	X

### Observations on known cancer genes

**Figure 3:**
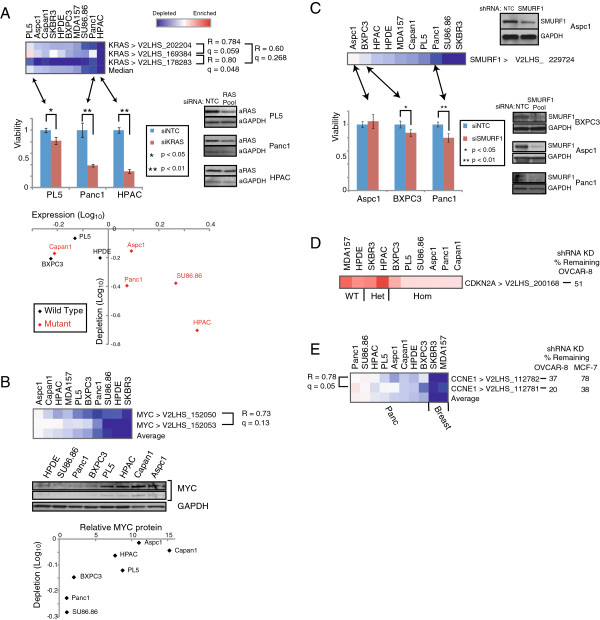
**Evaluation of known cancer genes. (A)***KRAS*. *Above left*, heatmap depicts shRNA depletion (blue) or enrichment (red) for each *KRAS*-targeting hairpin across the cell line panel (ordered by median shRNA depletion levels). The corresponding Pearson correlation (R) and false discovery rate (q) values are indicated. For select cell lines, the effect of KRAS knockdown (Western blot, *middle right*) on cell growth/viability (WST-1 assay, *middle left*) was independently verified by siRNA transfection. Indicated *P*-values determined by Student’s *t*-test (*KRAS*-siRNA *vs*. non-targeting control). *Below*, depletion of *KRAS*-targeting shRNAs is most evident in cell lines with *KRAS* activating mutation (red data points) and/or elevated KRAS transcript levels. *KRAS* mutational status was sequenced for PL5 [28] and previously reported for the other lines [18,29-32]. **(B)***MYC*. *Above*, heatmap shows shRNA depletion/enrichment profile, as described above. *Below*, levels of shRNA depletion are inversely correlated with MYC protein levels; MYC protein levels quantified by Western blot (*middle*). **(C)***SMURF1*. *Above left*, heatmap depicts shRNA depletion/enrichment profile; knockdown validated by Western blot (*middle*). For select cell lines, the effect of SMURF1 knockdown (Western blot, *below right*) on cell growth/viability (*below left*) was independently verified by siRNA transfection. **(D)***CDK2NA*. Heatmap depicts shRNA depletion/enrichment profile; *CDKN2A* deletion status of cell lines is indicated. Note, the shRNA used here was pre-validated [25] by Q-RT-PCR in OVCAR8 cell line (% residual transcript indicated). **(E)***CCNE1*. Heatmap depicts shRNA depletion/enrichment profile; shRNAs pre-validated (in OVCAR8 and MCF7 cells) as above.

Among the high-confidence shRNAs/phenotypes, several shRNAs targeting known oncogenes showed the anticipated pattern of depletion (i.e. knockdown reduces growth fitness) (Figure 3). For example, three different knockdown-validated [26] shRNAs targeting the *KRAS* oncogene were generally depleted across the panel of cell lines, though with some cell lines (e.g. Panc1 and HPAC) showing substantially more depletion than others (e.g. PL5) (Figure 3A). On-target knockdown of KRAS and consequent reduced cell viability were verified using independent *KRAS*-targeting siRNAs (Figure 3A). In pancreatic cancer, *KRAS* frequently harbors activating point mutations and may be amplified and/or overexpressed. Notably, in the cell line panel there was a good correlation of *KRAS*-shRNA screen depletion levels with both *KRAS* mutation status and *KRAS* transcript levels (R = 0.8). This pattern of dependency is consistent with classic oncogene addiction [27].

The shRNAs targeting the *MYC* oncogene also exhibited variable depletion across the panel of cell lines. However, unlike with *KRAS*, the cell lines with lower MYC transcript levels were associated with substantially higher depletion levels (Figure 3B). The basis for this observation is unclear, and we caution that the RNAi knockdown and phenotype requires validation. Nonetheless, we speculate that there might exist a threshold below which MYC levels are insufficient to support cell growth, and that in cell lines with high MYC levels incomplete shRNA-mediated knockdown is insufficient to pass that threshold.

We recently reported *SMURF1*, encoding an E3 ubiquitin ligase, to be amplified in a subset of pancreatic cancers, where it drives cell invasion but not growth in AsPC1 cells [33]. Here, in the context of the pooled shRNA screen, we reproduced that result. The single shRNA targeting *SMURF1* was neither depleted nor enriched in *SMURF1*-amplified AsPC1 cells (Figure 3C), consistent with its role in cell invasion but not growth. Interestingly, in non-amplified cell lines the *SMURF1* shRNA was generally depleted, suggesting a possible additional role in cell growth specific to a non-amplified context, a knockdown threshold effect as speculated above for *MYC* (endogenous SMURF1 expression levels are substantially elevated with amplification [33]), or an off-target effect. The findings for SMURF1 also suggest an added benefit of screening additional phenotypes, e.g. cell invasion.

We also observed the expected pattern of shRNA enrichment for shRNAs targeting known tumor suppressor genes, best exemplified by *CDKN2A*. A single shRNA targeting *CDKN2A* was enriched in a subset of the screened cell lines (Figure 3D). Notably, the cell lines that did not show substantial enrichment were in fact those with homozygous deletion of *CDKN2A* (where knockdown of a non-existent gene would not be expected to promote growth). These data suggest a utility of the shRNA knockdown screen approach in identifying not only oncogenes (by reduced growth fitness), but also potentially novel tumor suppressor genes (by enhanced growth fitness).

While the focus of our screen was pancreatic cancer, the inclusion of a breast cancer out-group also provided an opportunity to identify breast cancer selective dependencies. Among the high-confidence genes/phenotype, most notably shRNAs targeting the cell-cycle regulator *CCNE1* showed selective depletion in the two breast cancer cell lines (Figure 3E). CCNE1 has been reported to be the driver of 19q12 amplification in breast cancer [34,35]. Indeed, Natajaran *et al.* reported MDA157 breast cancer cells to be sensitive to CCNE1 knockdown [34], which we replicated here (Figure 3E). Our findings underscore a likely selective dependency and possible point of therapeutic attack in breast cancer.

### Cytoband 19q13 amplicon

**Figure 4:**
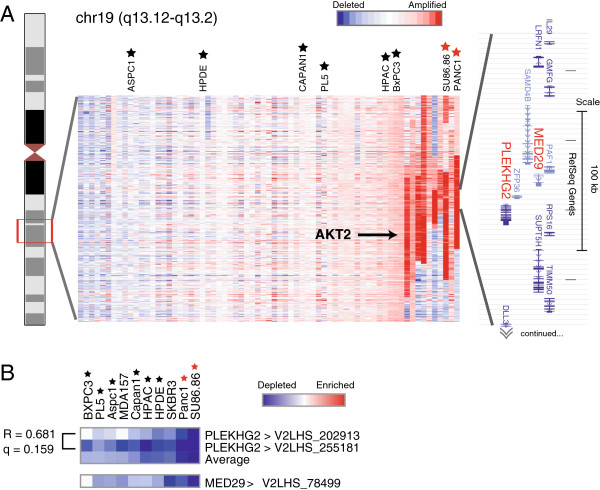
**Analysis of 19q13 amplicon genes. (A)** Heatmap representation of aCGH profiles [6] across the 19q13 region for a set of 70 pancreatic cancer cell lines and early-passage xenografts. Red intensity reflects DNA amplification (see key). Note, the smallest common region of amplification includes 12 genes (indicated) but excludes *AKT2* (labeled). Those pancreatic cancer cell lines also analyzed in the shRNA screen are identified by a star; those with 19q13 amplification are identified by a red star. Figure modified from [6]. **(B)** Heatmap depicts shRNA depletion/enrichment profiles for shRNAs targeting *PLEKHG2* and *MED29*. R and q values are indicated (see Figure 3 legend). Cell lines also included in the aCGH analysis are identified by a star; those with 19q13 amplification by a red star. Note that shRNA depletion levels are highest in pancreatic cell lines with 19q13 amplification.

While *KRAS* (12p12) and *MYC* (8q24) are among the most commonly amplified oncogenes, the 19q13 region also shows broad amplification in the majority of pancreatic cancers, and high-amplitude, focal amplifications in approximately 20% of cases (Figure 4A). The recurrence and focality strongly suggest the presence of an important oncogene within this amplicon. *AKT2*, which functions in the PI3 kinase pathway, resides near this chromosomal region, and would be a natural candidate for the primary driver of this amplification. However, as we previously noted [6], the smallest common region of amplification occurs proximal to *AKT2*, as *AKT2* is excluded from the amplicon in several pancreatic cancers (Figure 4A). These data imply that *AKT2* is not the principal driver of 19q13 amplification. To evaluate this amplicon, our screen included shRNAs targeting each of the 12 genes residing within the smallest common region of amplification.

From our screen, three shRNAs targeting genes residing at 19q13 were preferentially depleted in those cell lines harboring 19q13 amplification (Figure 4B). Among these, two high-confidence (correlated) shRNAs targeted *PLEKHG2*, encoding a plekstrin homology domain protein, and a single shRNA (and the only one included in the screen) targeted MED29, a mediator of RNA polymerase II transcription complex subunit. These data agree well with findings described by Kuuselo *et al.*[36]. Those investigators performed a focused siRNA-based analysis of genes within the 19q13 amplicon, reporting that knockdown of PLEKHG2 and MED29 reduced cell viability in Panc1 (19q-amplified) but not Miapaca2 (not amplified) cells. Thus, our data corroborate and extend (using independent assays and additional cell lines) prior work and highlight a still under-appreciated role of *PLEKHG2* and *MED29* (rather than AKT2) as likely oncogenes driving 19q13 amplification in pancreatic cancer.

### NUP153/FAST1 TGFβ signaling axis

**Figure 5:**
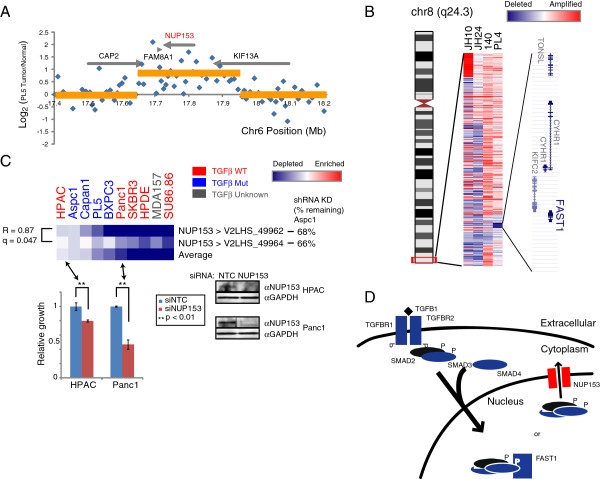
**Screen implicates NUP153-FAST1 SMAD shuttling axis. (A)** By aCGH, *NUP153* is found focally amplified in PL5 pancreatic cancer cells. Y-axis reports tumor/normal log_2_ copy number ratios, and x-axis reports Mb position along a region of chromosome 6. Yellow line depicts smoothed copy number calls (by circular binary segmentation). Genes are indicated by gray bars. The aCGH data are analyzed from [6]. **(B)** By aCGH, *FAST1* is focally deleted in several pancreatic cancers. In the heatmap, blue and red intensity reflect DNA deletion and amplification, respectfully. The smallest common region of deletion converges on five genes including *FAST1*. Note that FAST1 was not included among the 185 genes in the shRNA screen. **(C)** Screen profiles of *NUP153* shRNAs. *Above left*, heatmap depicts shRNA depletion (blue) for each *NUP153*-targeting shRNA across the cell line panel; R and q values are indicated (see Figure 3 legend). TGFβ pathway status (wildtype, mutant, or unknown) is indicated as reported [18,30,38] or empirically determined here (see Additional file 5). Note that depletion of *NUP153*-targeting shRNAs is most evident in cell lines with wildtype TGFβ signaling. The shRNAs used had been validated by Q-RT-PCR in the Aspc1 cell line (% residual transcript indicated). For select cell lines, the effect of NUP153 knockdown (Western blot, *below right*) on cell growth/viability was independently verified by siRNA transfection. P-values determined by Student’s *t*-test. **(D)** Proposed model illustrating a role of SMAD shuttling in pancreatic cancer. Blue gene-products are those gene identified to be deleted/mutated in pancreatic cancer and red gene-products as those amplified.

One novel observation derived from our screen centers on *NUP153*, which encodes a nuclear pore complex protein (or nucleoporin). *NUP153* was included in the screen because we identified it to be focally amplified in a single pancreatic cancer cell line, PL5 (Figure 5A). The NUP153 nucleoporin regulates the distribution of specific proteins between the nucleus and the cytoplasm, interestingly including the transducer of TGFβ signaling, SMAD2 [37]. In particular, NUP153 stoichiometrically competes with FAST1, a SMAD2 nuclear retention factor, to shuttle SMAD2 out of the nucleus, thus dampening TGFβ signaling. Further implicating this axis in pancreatic cancer, we also noted recurrent focal deletions of *FAST1* in other pancreatic cancer cases (Figure 5B).

In our screen, two different high-confidence shRNAs targeting *NUP153* showed varying levels of depletion across the cell line panel. Notably, cell lines with an intact upstream TGFβ signaling pathway (determined by absence of *SMAD4* and *TGFBR2* mutation/deletion, and/or TGFβ-induced growth suppression) generally exhibited higher levels of *NUP153*-shRNA depletion, compared to cell lines with a compromised TGFβ signaling pathway (Figure 5C) (Additional file 5). Our findings therefore suggest that altered NUP153-FAST1 shuttling of SMADs may provide an alternate means by which pancreatic cancers disrupt TGFβ signaling. The role of an altered NUP153/FAST1 TGFβ shuttling axis in pancreatic carcinogenesis warrants further investigation.

### KLF5 dependency

**Figure 6:**
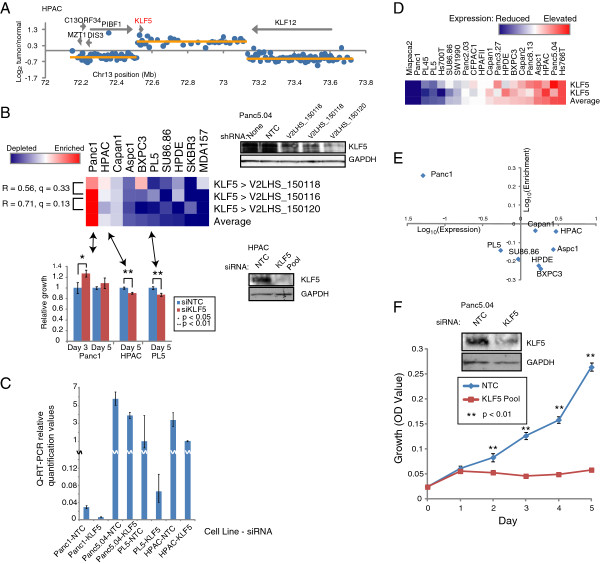
**An oncogenic role for KLF5. (A)** By aCGH, *KLF5* is found focally amplified in HPAC pancreatic cancer cells. Data plotted as described in Figure 4A legend. **(B)** Screen profiles of *KLF5* shRNAs. *Above left*, heatmap depicts shRNA depletion/enrichment (blue/red) for each *KLF5*-targeting shRNA across the cell line panel; R and q values are indicated (see Figure 3 legend). For select cell lines, Western-blot verification of KLF5 on-target knockdown was done by individual shRNA transfection (*above right*) or siRNA transfection (*below right*). Effect of knockdown on cell growth/viability was independently verified by siRNA transfection (*below left*); P-values by Student’s *t*-test. **(C)** KLF5 on-target siRNA knockdown also verified by Q-RT-PCR. **(D)** Relative transcript levels of KLF5, from microarray data [6], across 20 pancreatic cell lines; red and blue intensity reflects increased and decreased expression, respectively. **(E)** Plot of KLF5 relative transcript levels (x-axis) *vs*. average KLF5-targeted shRNA depletion/enrichment (y-axis). Note that most cell lines expressing KLF5 transcript are KLF5-dependent, except for Panc1 cells in which KLF5 transcript is barely detectable. **(F)** Knockdown of KLF5 (verified by Western, above) results in reduced growth/viability of Panc5.04 cells, heterozygous for the KLF5 (H389N) mutation. *P*-value by Student’s *t*-test (KLF5 siRNA *vs*. non-targeting control siRNA).

Another compelling finding relates to KLF5, the intestinal kruppel-like transcription factor. *KLF5* was included in our screen because we identified it to be focally amplified in a pancreatic cancer cell line, HPAC (Figure 6A). Interestingly, KLF5 has been paradoxically reported to function as both a tumor suppressor [39,40] and an oncogene [41,42] in different tumor types (reviewed in [43]). Our finding of focal *KLF5* amplification in pancreatic cancer suggests a possible oncogenic role.

In our screen, three different high-confidence shRNAs targeting *KLF5* showed depletion in most cell lines (Figure 6B). Though interestingly, a single cell line, Panc1, with extremely low levels of KLF5 transcript showed substantial *KLF5*-shRNA enrichment (Figure 6B-D). We verified on-target KLF5 knockdown and the contrasting knockdown growth-phenotypes (in Panc1 compared to two other lines) by siRNA transfection (Figure 6B, C). The basis for shRNA enrichment in the sole Panc1 line is unclear. We speculate that KLF5 might be epigenetically-silenced in Panc1 (based on barely-detectable expression) because in that cell line it plays a growth suppressive role; thus further knockdown would promote cell growth. In contrast, in every other cell line in the panel, KLF5 transcript levels are appreciable, and knockdown reduces cell growth (Figure 6D, E). Taken together, our findings support a context-dependency of KLF5 function, whilst the preponderance of data (focal amplification and growth dependency) supports a predominantly oncogenic role in pancreatic cancer.

Of note, a recent exome sequencing study [7] reported a single heterozygous mutation (H389N) in the DNA-binding domain of KLF5 in the pancreatic cell line Panc5.04. The authors interpreted the mutation to be functionally inactivating, suggesting a likely tumor suppressive role. Although Panc5.04 was not included in our screening panel, we sought to further characterize KLF5 in that line. By analysis of microarray data [6], KLF5 showed relatively high transcript levels in Panc5.04 compared to other pancreatic cancer cell lines (Figure 6D). Further, from our prior transcriptome sequencing (RNA-seq) data [6], 64% of KLF5 reads in Panc5.04 mapped to the wildtype allele (78 WT reads *vs*. 45 mutant reads), thus excluding epigenetic silencing of the wildtype allele. Notably, siRNA mediated knockdown of KLF5 in Panc5.04 cells resulted in marked growth inhibition (Figure 6F). This finding suggests that the single *KLF5* mutation in Panc5.04 is most likely a passenger mutation, and provides additional support for *KLF5* being predominantly oncogenic in pancreatic cancer. Future studies should define the transcriptional targets and mechanisms underlying KLF5 dependency in pancreatic cancer.

## Conclusions

In summary, we have detailed a proof-of-principle approach for the highly-parallel functional evaluation of candidate cancer genes, here for pancreatic cancer. We have simultaneously evaluated 185 candidate pancreatic cancer genes, selected as those recurrently and focally amplified or deleted, by a pooled shRNA library screen on 10 genetically-diverse cell lines. We have also described a novel approach, COPOTE, to enrich for on-target shRNAs and knockdown phenotypes, based on identifying shRNAs targeting the same gene that exhibit correlated phenotype. Our screen has uncovered novel pancreatic cancer genes and pathways, most notably highlighting potential roles of a putative NUP153-FAST1 SMAD shuttling axis controlling TGFβ signaling and an oncogenic function of the KLF5 transcription factor, both meriting further study. Future screens might include more shRNAs per gene (plausibly decreasing the likelihood of false negatives), more cell lines, and additional phenotypic assays. Nonetheless, our study here supports the general feasibility of a highly-parallel functional analysis of candidate cancer genes, addressing a fundamental bottleneck in the annotation of cancer genomes.

### Note added in proof

During review of our manuscript, Shao *et al.*[44] described an RNAi-screen analysis approach (ATARiS) that is conceptually similar to ours (COPOTE).

## Abbreviations

aCGH: Array-based comparative genomic hybridization; COPOTE: COrrelated phenotypes for on-target effects; FDR: False discovery rate; KD: Knockdown; PCR: Polymerase chain reaction; RNAi: RNA interference; RT-PCR: Reverse transcription PCR; Q-RT-PCR: Quantitative RT-PCR; shRNA: Short hairpin RNA; siRNA: Short interfering RNA.

## Competing interests

The authors declare that they have no competing interests.

## Authors’ contributions

AHS and JRP conceived the study, designed experiments, and wrote the manuscript. AHS and KS developed and optimized the screen. AHS performed the screen and all subsequent analysis and follow up experiments. CPG characterized the genetic context of various cell lines. All authors read and approved the manuscript.

## Supplementary Material

Additional file 1 Genes included in the shRNA screen. Click here for file

Additional file 2 **Depletion/enrichment (log**_**10**_** ratio) of each shRNA for each cell line (cell line replicates averaged).** Click here for file

Additional file 3 **Depletion/enrichment (log**_**10**_** ratio) of each shRNA for each cell line replicate screened.** Click here for file

Additional file 4 Correlation coefficients (R) and false discovery rates (q) for all hairpins targeting the same gene. Click here for file

Additional file 5 TGFβ pathway status, empirically determined by TGFβ-responsive growth inhibition, in pancreatic cancer cell lines (A) PL5 (unresponsive/mutant pathway) and (B) HPAC (responsive/wildtype pathway). Click here for file
