# Supplementary figures and images for: Integrative genomic and functional profiling of the pancreatic cancer genome
Source: BMC Genomics. 2013 Sep 16;14:624. doi: 10.1186/1471-2164-14-624 (PMC3848637; doi:10.1186/1471-2164-14-624)

# Additional File 5

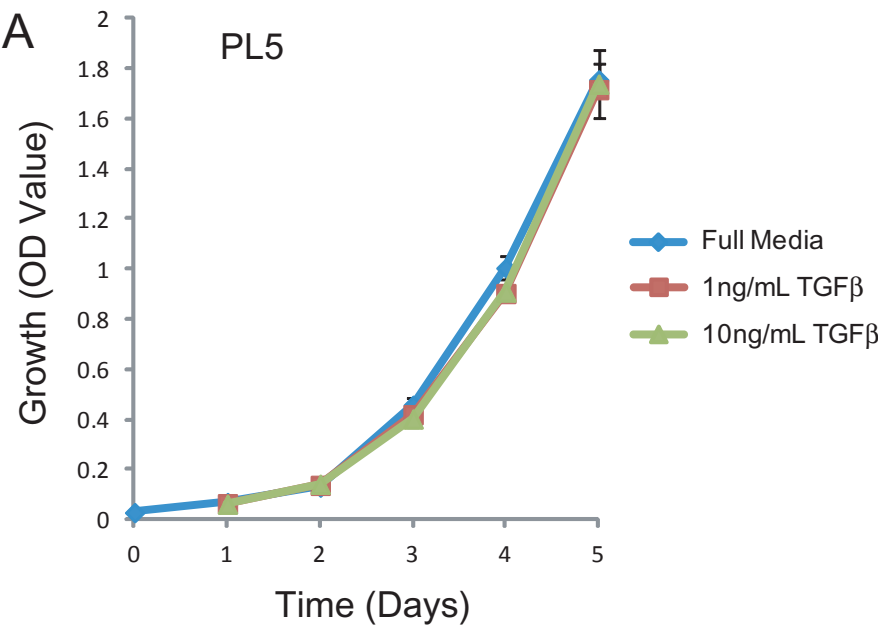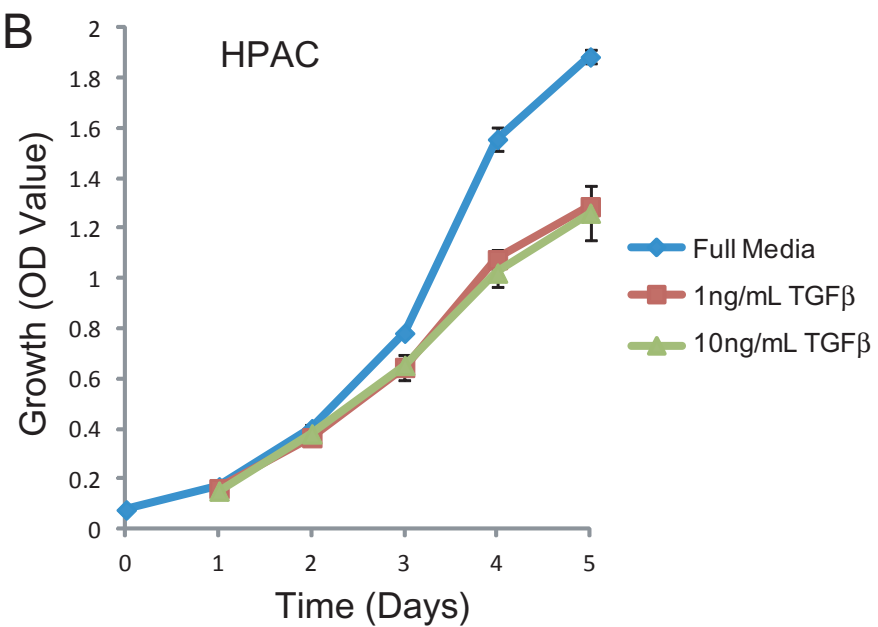

Supplement: Additional file 5 — TGFβ pathway status, empirically determined by TGFβ-responsive growth inhibition, in pancreatic cancer cell lines (A) PL5 (unresponsive/mutant pathway) and (B) HPAC (responsive/wildtype pathway). [file 1471-2164-14-624-S5.pdf]
